# Burden and Risk Factors for Severe Respiratory Syncytial Virus Infection among Pediatric Hematopoietic Cell Transplant Recipients

**DOI:** 10.1093/jpids/piag092

**Published:** 2026-09-10

**Authors:** Diego R Hijano, Surabhi B Vora, Yilun Sun, Li Tang, Ronald H Dallas, Jose A Ferrolino, Madeline B Johnson, Kim J Allison, Monica Ardura, Emily Ansusinha, Juri Boguniewicz, Janine Dailey Garnes, Kathryn P Goggin, Alexis Gossett, Benjamin Hanisch, Nour Hasan, Sarah M Heston, Jennifer R Jackson, Madam Kumar, Courtney McDonald, Eric McGrath, William J Muller, Anne Rose, Frances Saccoccio, Beth K Thielen, Mary Suzanne Whitworth, Lara Danziger-Isakov, Janet A Englund, Gabriela Maron, Chikara Ogimi

**Affiliations:** Department of Infectious Diseases, St. Jude Children’s Research Hospital, Memphis, TN, United States; Department of Pediatrics, University of Tennessee Health Science Center, Memphis, TN, United States; Division of Pediatric Infectious Diseases, Seattle Children’s Research Institute, University of Washington, Seattle, WA, United States; Department of Biostatistics, St. Jude Children’s Research Hospital, Memphis, TN, United States; Department of Biostatistics, Cleveland Clinic, Cleveland, OH, United States; Department of Infectious Diseases, St. Jude Children’s Research Hospital, Memphis, TN, United States; Department of Infectious Diseases, St. Jude Children’s Research Hospital, Memphis, TN, United States; Department of Infectious Diseases, St. Jude Children’s Research Hospital, Memphis, TN, United States; Department of Infectious Diseases, St. Jude Children’s Research Hospital, Memphis, TN, United States; Department of Infectious Diseases, Nationwide Children’s Hospital, Columbus, OH, United States; Department of Infectious Diseases, Children’s National Hospital, Washington, DC, United States; Department of Pediatrics, University of Colorado School of Medicine, Aurora, CO, United States; Department of Infectious Diseases, Infection Control and Employee Health, The University of Texas MD Anderson Cancer Center, Houston, TX, United States; Division of Infectious Diseases, Department of Pediatrics, Emory University and Children’s Healthcare of Atlanta, Atlanta, GA, United States; Department of Pediatrics, Cook Children’s Hospital, Fort Worth, TX, United States; Department of Infectious Diseases, Children’s National Hospital, Washington, DC, United States; Division of Pediatric Infectious Disease, Department of Pediatrics, University of Alabama at Birmingham, Birmingham, AL, United States; Department of Pediatrics, Duke University School of Medicine, Durham, NC, United States; Department of Infectious Diseases, Infection Control and Employee Health, The University of Texas MD Anderson Cancer Center, Houston, TX, United States; Department of Pediatrics, UChicago Medicine, Chicago, IL United States; Department of Obstetrics and Gynecology, Intermountain Health, St. Joseph Hospital, Denver, CO, United States; Department of Infectious Diseases, Wayne State University School of Medicine, Detroit, MI, United States; Ann and Robert H. Lurie Children’s Hospital of Chicago, Stanley Manne Children’s Research Institute, Chicago, IL, United States; Department of Pediatrics, University of Cincinnati College of Medicine, Cincinnati Children’s Hospital Medical Center, Cincinnati, OH, United States; Department of Infectious Diseases, UF Health Shands Children’s Hospital, Gainesville, FL, USA; Division of Pediatric Infectious Diseases, Department of Pediatrics, University of Minnesota Medical School, Minneapolis, MN, United States; Department of Pediatrics, Cook Children’s Hospital, Fort Worth, TX, United States; Department of Pediatrics, University of Cincinnati College of Medicine, Cincinnati Children’s Hospital Medical Center, Cincinnati, OH, United States; Division of Pediatric Infectious Diseases, Seattle Children’s Research Institute, University of Washington, Seattle, WA, United States; Department of Infectious Diseases, St. Jude Children’s Research Hospital, Memphis, TN, United States; Division of Infectious Diseases, National Center for Child Health and Development, Tokyo, Japan

**Keywords:** respiratory syncytial virus (RSV), Pediatric hematopoietic cell transplant (HCT), immunocompromised children, RSV outcomes, risk factors

## Abstract

**Background:**

Respiratory syncytial virus (RSV) causes substantial morbidity in pediatric hematopoietic cell transplant (HCT) recipients; however, risk factors for severe disease remain incompletely characterized.

**Methods:**

We conducted a multicenter retrospective cohort study across 16 U.S. transplant centers, evaluating children and adolescents (0–19 years) who underwent HCT and developed a laboratory-confirmed RSV infection between 2010 and 2019. Clinical characteristics, laboratory values, and immunosuppressive and transplant history were analyzed. Outcomes included new or increased supplemental oxygen requirement, RSV-related hospitalization, progression to lower respiratory tract infection (LRTI), invasive mechanical ventilation, and 90-day mortality. Univariate logistic regression was used to screen candidate predictors, followed by the least absolute shrinkage and selection operator for variable selection, and multivariable logistic regression to identify independent predictors associated with clinical outcomes.

**Results:**

Among 204 pediatric HCT recipients with RSV infection, 41.7% had RSV-related hospitalization, 21.2% required new or increased supplemental oxygen, and 4% required invasive mechanical ventilation within 28 days after the diagnosis. Mortality within 90 days was 5.4%, with one death attributable to RSV. In multivariable models, female sex, younger age (particularly <5 years), neutropenia (ANC <500/mm^3^), presence of co-pathogen in the respiratory tract and chronic lung disease were independently associated with this oxygen outcome, while non-peripheral graft sources and T-cell–depleting therapy were associated with RSV-related hospitalization.

**Conclusions:**

Severe outcomes are common in pediatric HCT recipients with RSV infection. Younger age, markers of impaired immune recovery, history of chronic lung disease, and T-cell–depleting therapy are major determinants of adverse outcomes. These findings support targeted risk stratification and may inform preventive and treatment strategies in this high-risk population.

## INTRODUCTION

Respiratory syncytial virus (RSV) is the leading cause of lower respiratory tract infection (LRTI) in young children worldwide.^1–4^ In immunocompromised hosts, including pediatric hematopoietic cell transplant (HCT) recipients, RSV can cause substantial morbidity with increased risks of pneumonia, respiratory failure, and death compared with otherwise healthy children.^5–7^

Prior studies in adult HCT recipients identified risk factors for poor outcomes and led to validated clinical scoring systems that guide prognostication and management in that population.^8^^,^^9^ In contrast, there are not comparable multicenter data to define risk factors for severe RSV disease in pediatric HCT recipients or to support development of pediatric-specific prediction tools. Existing pediatric literature is limited by small cohorts, single-center designs, and incomplete representation of transplant characteristics and immune status.^10–12^

The need for improved risk stratification in children is particularly pressing. Treatment options remain limited; ribavirin is the only licensed antiviral, yet its optimal dosing in children is uncertain, and inhaled formulations are costly and logistically challenging.^13–20^ RSV prophylaxis guidelines from the American Academy of Pediatrics do not specifically address HCT recipients.^21^^,^^22^ Although long-acting monoclonal antibodies such as nirsevimab and clesrovimab expand preventive options for young children, including immunocompromised hosts, their effectiveness, dosing, and tolerability for older children are unknown. Furthermore, three RSV vaccines are currently licensed in the United States, none of which is available for children.^23^^,^^24^ As new preventive and therapeutic strategies continue to advance, defining which immunocompromised children stand to benefit most becomes increasingly important, including identifying populations in whom the risk–benefit balance may favor participation in clinical trials of investigational antivirals.^25–27^

To address these gaps, we conducted a multicenter retrospective study across U.S. pediatric transplant centers to characterize the risk factors for morbidity and mortality associated with RSV infection in pediatric HCT recipients. We evaluated clinical, transplant-related, and immunologic determinants of disease severity, including RSV-related hospitalization, new or increased supplemental oxygen requirement, LRTI, invasive mechanical ventilation, and mortality.

## METHODS

### Study Design and Cohort

We conducted a multicenter retrospective observational cohort study coordinated by the Pediatric Infectious Diseases Transplant Network (PIDTRAN) at St. Jude Children’s Research Hospital. The study was approved by the Institutional Review Board (IRB) at St. Jude and by each participating institution, with a waiver of informed consent.

Children and adolescents were eligible if they met the following criteria: (1) underwent HCT between January 1, 2010 and December 31, 2019; (2) were younger than 20 years at the time of transplant; and (3) had a first RSV infection identified within 365 days post-transplant. Data from 16 U.S. pediatric transplant centers were included.

### Study Definitions

RSV infection was defined as the presence of respiratory symptoms along with laboratory confirmation of RSV by a clinically validated diagnostic test. LRTI was defined as RSV detected from a lower respiratory tract specimen, such as tracheal aspirate or bronchoalveolar lavage, or as RSV detected from an upper respiratory tract specimen accompanied by new radiographic abnormalities consistent with LRTI and a clinician’s diagnosis of bronchiolitis or pneumonia. RSV-related hospitalization was recorded in the case report form as a predefined study outcome within 28 days after RSV diagnosis. No additional coding or adjudication was applied for patients already hospitalized at the time of diagnosis. Supplemental oxygen requirement was defined as new or increased oxygen support above baseline delivered by any modality and sustained for more than 4 hours per day. Lymphopenia, neutropenia, and monocytopenia were defined a priori as ALC <300/μL, ANC <500/mm^3^, and absolute monocyte count <100/μL, respectively, based on prior HCT respiratory virus literature and expert consensus within the study group. Organ failure at diagnosis was defined as documented dysfunction of one or more non-respiratory organ systems (eg, renal, hepatic, cardiovascular, or neurologic) present at the time of RSV diagnosis, as recorded in the medical record. Comorbidities were recorded as a binary variable indicating the presence of one or more clinician-documented conditions captured in the REDCap case report form, including heart, kidney, or liver failure; multiorgan failure; pulmonary disorders; or another specified condition. Chronic lung disease was also captured and analyzed as a separate prespecified variable. Respiratory co-pathogens included concomitant viral, bacterial, or fungal pathogens identified from respiratory specimens, while blood co-pathogens included viral DNAemia/viremia and bacterial or fungal bloodstream pathogens documented in the medical record. Formal adjudication of pathogen significance was not performed. Given the retrospective multicenter design, standardized physiologic thresholds were not applied, and all definitions were based on clinician documentation recorded in the medical record.

### Outcomes of Interest

The primary objective of the study was to characterize the clinical outcomes and severity of RSV infection in pediatric HCT recipients using outcomes previously described in the literature and of established clinical relevance.^27–30^ These outcomes included new or increased supplemental oxygen requirement above baseline (primary outcome), RSV-related hospitalization assessed within 28 days after RSV diagnosis, development of LRTI and requirement for invasive mechanical ventilation, each assessed within 28 days after RSV diagnosis, and RSV-attributable death assessed within 90 days of diagnosis. The oxygen outcome and LRTI were evaluated as separate clinical outcomes according to the prespecified study definitions.

### Data Collection

Clinical data were abstracted from the EMR into the REDCap system hosted at the PIDTRAN Coordinating Center at St. Jude. Cases were identified at each participating site by applying standardized study inclusion criteria to capture all eligible patients with laboratory-confirmed RSV infection during the study period. Collected variables included demographics, transplant characteristics, underlying diagnoses, comorbidities, graft-versus-host disease, receipt of immunosuppressive and antiviral therapies, palivizumab (the only RSV-specific monoclonal antibody available during the study period), immune globulin, and laboratory results, selected a priori based on prior HCT respiratory virus studies, including those conducted by PIDTRAN.^31^^,^^32^

### Data Analysis

Continuous variables were summarized as medians and ranges and compared using the Wilcoxon–Mann–Whitney test. Categorical variables were reported as counts and percentages and compared using Fisher’s exact test.

Risk factors for each clinical outcome were evaluated using univariate and multivariable logistic regression with robust standard errors. Robust standard errors were used to reduce reliance on conventional model-based standard error estimates, particularly because some models included sparse covariate patterns that could result in high-leverage observations. Variables with a *P*-value < .10 in univariate analyses were included in a candidate pool and entered into least absolute shrinkage and selection operator (LASSO) models for variable selection. This initial screening step was used to reduce the number of candidate predictors considered in LASSO, given the limited number of events for some outcomes and missing data among candidate predictors. The LASSO penalty parameter was selected using 10-fold cross-validation, with cross-validated ROC AUC used as the selection criterion. Variables retained by LASSO were included in the final multivariable logistic regression models, regardless of their individual *p*-values. Statistical analyses were performed using R version 4.5.1, and a two-sided *p*-value <0.05 was considered statistically significant.

## RESULTS

### Cohort Characteristics

A total of 204 pediatric HCT recipients with RSV infection were included. The median age at diagnosis was 6 years (range, 0–19), and 10.8% of patients were younger than 2 years. Males accounted for 54% of the cohort and 58% were White. Hematologic malignancies were the largest single underlying indication for transplantation (42.2%). Most children (84.8%) had undergone a single transplant, and 77% underwent allogeneic HCT. Myeloablative conditioning was used in 73% of recipients. T-cell–depleting agents (ATG or alemtuzumab) were included in conditioning regimens for 27% of patients (Table 1).

**Table 1:** Demographic and Clinical Characteristics of Pediatric HCT Recipients with and Without Oxygen Requirement at First RSV Infection

Variable	Total (N = 204)	Yes (Oxygen) N = 43	No (Oxygen) N = 160	*P* value
Age (y), median [min, max]	6.0 [0-19.0]	4.0 [0.0–18.0]	7.0 [0.0–19.0]	.009
Age group, n (%)				.024
<2 y	22 (10.8%)	8 (18.6%)	14 (8.8%)	
2–4 y	53 (26.0%)	15 (34.9%)	37 (23.1%)	
≥5 y	129 (63.2%)	20 (46.5%)	109 (68.1%)	
Sex, n (%)				.085
Male	111 (54.4%)	18 (41.9%)	92 (57.5%)	
Female	93 (45.6%)	25 (58.1%)	68 (42.5%)	
Race, n (%)				.39
White	119 (58.3%)	28 (65.1%)	91 (56.9%)	
Other or unknown	85 (41.7%)	15 (34.9%)	69 (43.1%)	
Underlying diagnosis				.002
Hematologic malignancy only	86 (42.2%)	9 (20.9%)	77 (48.1%)	
Others^	118 (57.8%)	34 (79.1%)	83 (51.9%)	
Myeloablative conditioning, n (%)	149 (73.0%)	35 (81.4%)	113 (70.6%)	.18
Chronic lung disease, n (%)	17 (8.5%)	7 (16.7%)	10 (6.4%)	.057
T-cell–depleting induction (ATG or alemtuzumab), n (%)	55 (27.0%)	17 (39.5%)	38 (23.8%)	.052
B-cell depletion ≤6 months before RSV, n (%)	18 (8.9%)	1 (2.3%)	17 (10.7%)	.13
CMV serostatus, n (%)				.34
D+/R+	54 (32.5%)	13 (36.1%)	41 (31.5%)	
D+/R−	14 (8.4%)	3 (8.3%)	11 (8.5%)	
D−/R+	31 (18.7%)	3 (8.3%)	28 (21.5%)	
D−/R−	67 (40.4%)	17 (47.2%)	50 (38.5%)	
Number of transplantations, n (%)				.15
1	173 (84.8%)	33 (76.7%)	139 (86.9%)	
2–3	31 (15.2%)	10 (23.3%)	21 (13.1%)	
HCT type, n (%)				.10
Autologous	47 (23.0%)	14 (32.6%)	32 (20.0%)	
Allogeneic	157 (77.0%)	29 (67.4%)	128 (80.0%)	
Source of transplant				.95
Cord blood	39 (19.1%)	9 (20.9%)	30 (18.8%)	
Bone marrow	101 (49.5%)	21 (48.8%)	80 (50.0%)	
Peripheral blood	64 (31.4%)	13 (30.2%)	50 (31.3%)	
Days from transplant to RSV diagnosis, median [range]	153 [0–365]	140 [0-365]	161 [2-365]	.024
GvHD prophylaxis or treatment at RSV diagnosis, n (%)	114 (55.9%)	21 (48.8%)	93 (58.1%)	.30
Organ failure at RSV diagnosis, n (%)	11 (5.4%)	4 (9.3%)	7 (4.4%)	.25
Steroids ≤2 weeks before RSV diagnosis, n (%)	62 (30.4%)	14 (32.6%)	48 (30.0%)	.85
IVIG ≤4 weeks before RSV diagnosis, n (%)	72 (35.3%)	20 (46.5%)	52 (32.5%)	.11
Palivizumab ≤4 weeks before RSV diagnosis, n (%)	8 (3.9%)	1 (2.3%)	7 (4.4%)	> .99
Co-pathogen in respiratory tract, n (%)	63 (30.9%)	18 (41.9%)	45 (28.1%)	.10
Co-pathogen in blood, n (%)	25 (12.3%)	6 (14.0%)	19 (11.9%)	.79
Lowest ALC <300/μL before RSV, n (%)	51 (25.6%)	17 (40.5%)	34 (21.8%)	.018
Closest ALC <300/μL to RSV, n (%)	40 (19.9%)	17 (40.5%)	23 (14.6%)	< .001
Lowest ANC <500/mm^3^ before RSV, n (%)	34 (16.9%)	15 (35.7%)	19 (12.0%)	< .001
Closest ANC <500/mm^3^ to RSV, n (%)	24 (11.9%)	13 (31.0%)	11 (6.9%)	< .001
Lowest monocyte count <100/μL before RSV, n (%)	38 (19.1%)	12 (28.6%)	26 (16.7%)	.12
Closest monocyte count <100/μL to RSV, n (%)	30 (14.9%)	13 (31.0%)	17 (10.8%)	.003

^ Others include brain tumor (8), solid tumor (29), hematologic disorder (33), inherited immunodeficiency (23), other underlying diagnosis (18), and multiple conditions (7).

Abbreviations: ALC, absolute lymphocyte count; ANC, absolute neutrophil count; ATG, antithymocyte globulin; CMV, cytomegalovirus; D, donor; GvHD, graft-versus-host disease; HCT, hematopoietic cell transplantation; IVIG, intravenous immunoglobulin; R, recipient; RSV, respiratory syncytial virus.

At the time of RSV diagnosis, 55.9% of children were receiving GVHD prophylaxis or treatment. Co-pathogens were identified within 48 hours of RSV detection in 30.9% of children from respiratory samples and 12.3% from blood samples; detailed distributions of respiratory and blood co-pathogens are provided in Supplementary Table 1. Steroid exposure within the preceding 2 weeks was found in 30.4% of the cohort. IVIG and palivizumab had been administered within 4 weeks prior to RSV diagnosis in 35.3% and 3.9% of patients, respectively. Organ failure at diagnosis was uncommon (5.4%) (Table 1). Ribavirin was administered to 49 (22.9%) patients. IVIG and palivizumab were administered within 28 days after RSV diagnosis in 90 (42.1%) and 34 (15.9%) patients, respectively. Because these therapies were administered after diagnosis and frequently in response to clinical severity, they were not included in the primary multivariable risk factor models to minimize confounding by indication.

### Clinical Outcomes

Of the 204 children, 85 (41.7%) had RSV-related hospitalization, and 43 (21.2%) required new or increased supplemental oxygen during their illness. Of these 43 children, 18 (41.9%) had a baseline supplemental oxygen requirement before RSV diagnosis. Twenty-eight children (13.7%) presented with LRTI at diagnosis. Among the 176 patients who presented with URTI at diagnosis, progression status was available for 172, of whom 10 (5.8%) subsequently progressed to LRTI. Invasive mechanical ventilation was required in eight children (3.9%). Among these 43 children, the maximum level of respiratory support was low-flow oxygen in 26 (60.5%), high-flow nasal cannula in 6 (14.0%), CPAP/BiPAP in 1 (2.3%), and invasive mechanical ventilation in 8 (18.6%); support level could not be determined for 2 children (Supplementary Table 2). Eleven children (5.4%) died within 90 days of RSV diagnosis (median time from infection to death, 40 days; range, 11–68), with one death (0.5%) attributed directly to RSV (Figure 1).

**Figure 1:**
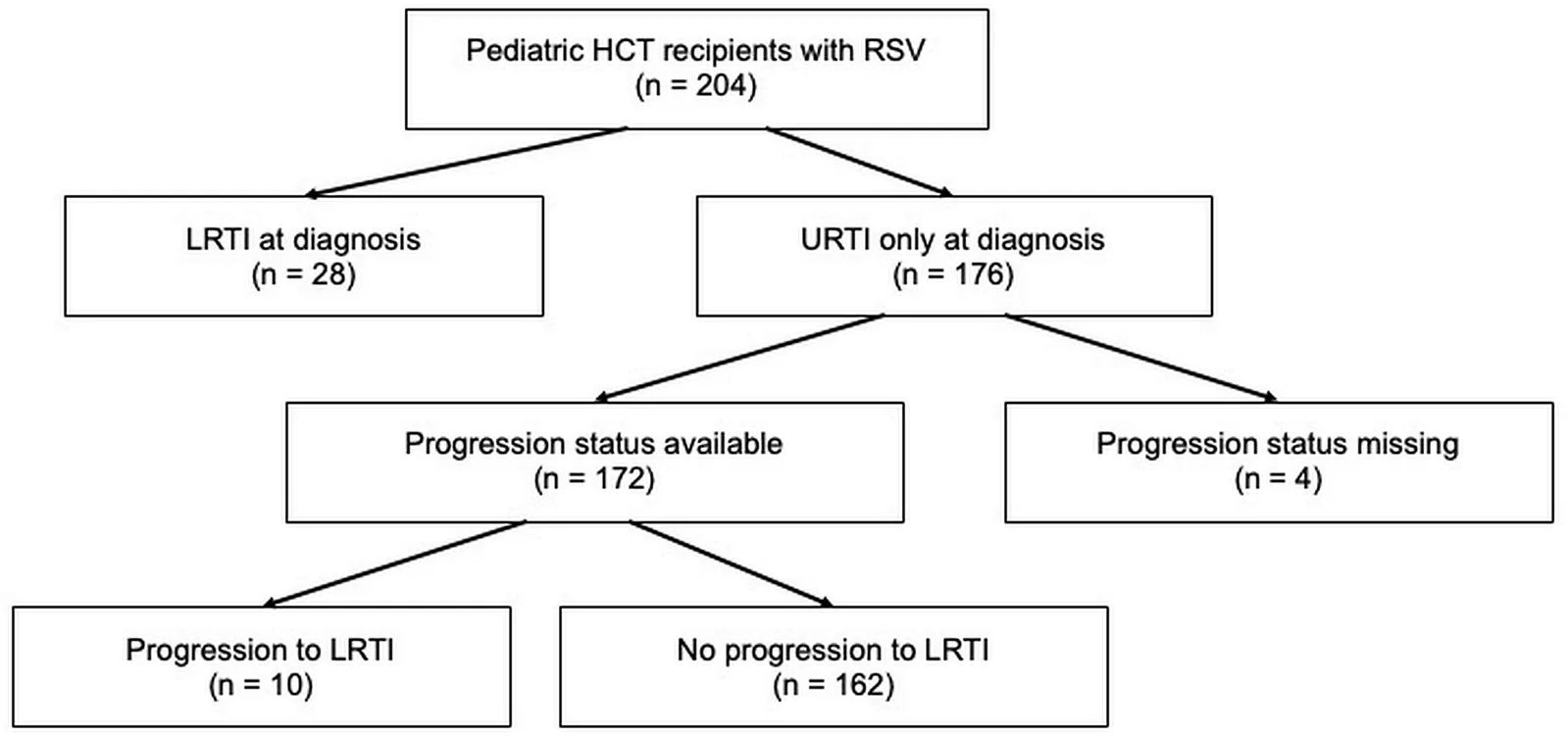
Among 204 Pediatric HCT Recipients with Laboratory-Confirmed RSV Infection, 28 (13.7%) Had LRTI at the Time of Diagnosis and 176 (86.3%) Presented with Upper Respiratory Tract Infection (URTI) Only. Among those with URTI at Diagnosis, Progression Status was Available for 172 Patients, of Whom 10 (5.8%) Progressed to LRTI and 162 (94.2%) Did Not Progress. Progression Status was Unavailable for 4 Patients

Children requiring additional oxygen support above baseline were younger and had more profound cytopenias than those who did not (Table 1). Similar patterns were observed for invasive mechanical ventilation and death, although event numbers were limited (Supplementary Table 3). Deaths occurred predominantly among children with comorbidities and organ failure (Supplementary Table 4).

### Risk Factors for Severe RSV Disease

#### New or Increased Supplemental Oxygen Requirement

In univariate analysis, younger age, shorter time from transplant to RSV diagnosis, exposure to ATG/Alemtuzumab, comorbidities, lower absolute lymphocyte counts (ALC), lower absolute neutrophil counts (ANC), and lower monocyte counts were all associated with new or increased supplemental oxygen requirement (Table 1). Table 2 shows two separate multivariable analyses using the lowest immune cell counts and the counts closest to RSV infection, in Multivariable Models 1 and 2, respectively. Neutropenia (ANC <500) was independently associated with this outcome (adjusted ORs 8.13 [*P* < .001] and 7.31 [*P* = .020], respectively). Although shorter time from transplant and lower ALC and monocyte counts were associated with this outcome in univariate analyses, none of these variables were retained in the final multivariable models following LASSO selection. Using children aged 2–4 years as the reference group, age ≥ 5 years was independently associated with lower odds of the oxygen outcome, whereas children <2 years had similar risk compared with those aged 2–4 years, indicating that the reduction in risk becomes apparent after 5 years of age. This protective association for children aged ≥5 years was consistent in a dichotomous analysis (<5 vs ≥5 years), in which age ≥ 5 years remained independently associated with lower odds of this outcome in both multivariable models (Supplementary Table 5). Female sex was independently associated with the oxygen outcome (adjusted ORs 3.82 and 3.67, *P* = .010 and .015), and chronic lung disease was also independently associated (adjusted ORs 4.23 [*P* = .033] and 4.33 [*P* = .024]). Co-pathogen detection in the respiratory tract was associated with this outcome in the lowest-cell-count model only (adjusted OR 2.38, *P* = .046). In the closest-cell-count model, low lymphocyte count (<300) was associated with this outcome in univariate analysis but was not retained after LASSO selection.

**Table 2:** Logistic Regression Analysis for Risk Factors Associated with Supplemental Oxygen Requirement Among HCT Recipients

	Univariate		Multivariable 1		Multivariable 2	
Variable	Raw OR (95% CI)	*P*-value	Adjusted OR (95% CI)	*P*-value	Adjusted OR (95% CI)	*P*-value
Age < 2y vs. 2-4y	1.41 (0.46, 4.29)	.546	0.90 (0.21, 3.80)	.887	0.95 (0.23, 4.03)	.949
Age ≥ 5y vs. 2-4y	0.45 (0.21, 0.98)	.044	0.35 (0.14, 0.88)	.025	0.35 (0.14, 0.90)	.030
Female vs. Male	1.88 (0.95, 3.73)	.071	3.82 (1.38, 10.60)	.010	3.67 (1.28, 10.49)	.015
Allogeneic (matched) vs. Autologous	0.51 (0.22, 1.17)	.112				
Allogeneic (other) vs. Autologous	0.53 (0.21, 1.30)	.163				
Peripheral Blood vs. Others	0.95 (0.46, 2.00)	.899				
ATG/Alemtuzumab vs. No/Other	2.10 (1.02, 4.31)	.044				
CMV status: R+ vs R-	0.80 (0.39, 1.63)	.536				
Number of transplantations (2 or 3 vs. 1)	2.01 (0.83, 4.84)	.121				
Co-morbidities (Yes vs. No)	2.03 (1.01, 4.06)	.045	1.93 (0.78, 4.75)	0.152	2.20 (0.86, 5.61)	0.098
Chronic lung disease (Yes vs. No)	2.92 (0.92, 9.23)	.068	4.23 (1.12, 15.99)	0.033	4.33 (1.21, 15.51)	0.024
GvHD (Yes vs. No)	0.90 (0.42, 1.92)	.792				
IVIG 4 weeks before RSV (Yes vs. No)	1.81 (0.91, 3.60)	.093				
Palivizumab 4 weeks before RSV (Yes vs. No)	0.52 (0.03, 8.21)	.643				
Steroids 2 weeks prior to RSV (Yes vs. No)	1.13 (0.54, 2.34)	.749				
Co-pathogen in blood (Yes vs. No)	1.20 (0.42, 3.47)	.732				
Co-pathogen in respiratory tract (Yes vs. No)	1.84 (0.91, 3.72)	.089	2.38 (1.02, 5.56)	0.046		
Days from transplant to RSV diagnosis	0.996 (0.993, 0.999)	.018				
Lowest ALC prior to RSV diagnosis (<300 μL vs. ≥300 μL)	2.44 (1.17, 5.08)	.017				
Lowest ANC prior to RSV diagnosis (<500 mm3 vs. ≥500 mm3)	4.06 (1.79, 9.22)	< .001	8.13 (2.65, 24.92)	<0.001		
Lowest monocyte prior to RSV diagnosis (<100 μL vs. ≥100 μL)	2.00 (0.89, 4.51)	.094				
Closest ALC prior to RSV diagnosis (<300 μL vs. ≥300 μL)	3.99 (1.84, 8.67)	< .001			2.61 (0.81, 8.43)	0.108
Closest ANC prior to RSV diagnosis (<500 mm3 vs. ≥500 mm3)	6.03 (2.31, 15.77)	< .001			7.31 (1.36, 39.23)	0.020
Closest monocyte prior to RSV diagnosis (<100 μL vs. ≥100 μL)	3.72 (1.57, 8.81)	.003				

Abbreviations: ALC, absolute lymphocyte count; ANC, absolute neutrophil count; ATG, antithymocyte globulin; CI, confidence interval; CMV, cytomegalovirus; GvHD, graft-versus-host disease; IVIG, intravenous immunoglobulin; LASSO, least absolute shrinkage and selection operator; OR, odds ratio; RSV, respiratory syncytial virus.

**MV1:** Multivariable model using the lowest immune cell count prior to RSV diagnosis. **MV2:** Multivariable model using the immune cell count closest to RSV diagnosis. These models represent two prespecified approaches to evaluating immune reconstitution.

Variables with a *P*-value < .10 in univariate analyses were included in the candidate pool for multivariable modeling, and final variable selection was performed using LASSO. Robust standard errors were computed using the sandwich estimator. A two-sided *p*-value <0.05 was considered statistically significant.

#### Hospitalization and LRTI

RSV-related hospitalization was recorded for 85 (41.7%) patients, although rates varied across institutions (Supplementary Table 6). In univariate analyses (Supplementary Table 7), this outcome was more frequent among children with comorbidities or chronic lung disease and among those who received ATG as part of transplant conditioning.

In both multivariable analyses using the lowest and closest cell counts (Supplementary Table 8), prior ATG/Alemtuzumab exposure was independently associated with higher odds of RSV-related hospitalization (adjusted OR 2.53, *P* = .008). In contrast, peripheral blood as the graft source was associated with lower odds of this outcome (adjusted OR 0.36, *P* = .006) in both models. Other factors that were significant in univariate analysis did not enter the final multivariable models.

Progression from URTI to LRTI was infrequent (*n* = 10), limiting us to univariate analyses (Table 3; Supplementary Table 9) and none of the factors were significant. In univariable analyses using a combined LRTI outcome (LRTI at diagnosis or progression from URTI to LRTI), children with LRTI were more likely to have nonmalignant underlying diagnoses, recent exposure to ATG or B cell depleting therapy, and lower lymphocyte counts before RSV diagnosis. However, in multivariable analyses (Supplementary Table 10), no factors remained statistically significant.

**Table 3:** Progression from URTI to LRTI Among HCT Recipients

Characteristic	Yes (N = 10)	No (N = 162)	*P*-value
Age group, n (%)			.42
<2y	0 (0.0%)	19 (11.7%)	
2-4y	4 (40.0%)	38 (23.5%)	
≥5y	6 (60.0%)	105 (64.8%)	
Sex, n (%)			.75
Male	6 (60.0%)	84 (51.9%)	
Female	4 (40.0%)	78 (48.1%)	
Number of transplantations, n (%)			.21
1	7 (70.0%)	137 (84.6%)	
2 or 3	3 (30.0%)	25 (15.4%)	
Source of transplant			.31
Cord blood	0 (0.0%)	31 (19.1%)	
Bone marrow	7 (70.0%)	79 (48.8%)	
Peripheral blood	3 (30.0%)	52 (32.1%)	
Allo-HCT type, n (%)			.39
Matched sibling donor	2 (28.6%)	32 (25.4%)	
Matched unrelated donor	4 (57.1%)	41 (32.5%)	
Mismatched unrelated donor	1 (14.3%)	7 (5.6%)	
Haploidentical	0 (0.0%)	19 (15.1%)	
Cord/related or unrelated	0 (0.0%)	25 (19.8%)	
Other	0 (0.0%)	2 (1.6%)	
Induction medications for transplantation, n (%)			.10
ATG	4 (40.0%)	26 (16.0%)	
Alemtuzumab	1 (10.0%)	13 (8.0%)	
No/Other	5 (50.0%)	123 (75.9%)	
CMV serostatus, n (%)			.69
D+/R+	3 (33.3%)	41 (31.5%)	
D+/R−	0 (0.0%)	12 (9.2%)	
D−/R+	3 (33.3%)	25 (19.2%)	
D−/R−	3 (33.3%)	52 (40.0%)	
Co-morbidities, n (%)			.077
Yes	6 (60.0%)	49 (30.2%)	
No	4 (40.0%)	113 (69.8%)	
Chronic lung disease, n (%)			> .99
Yes	1 (10.0%)	15 (9.5%)	
No	9 (90.0%)	143 (90.5%)	
GvHD diagnosis, n (%)			.87
None	7 (70.0%)	116 (71.6%)	
Acute	2 (20.0%)	33 (20.4%)	
Chronic	1 (10.0%)	13 (8.0%)	
Both-Acute and Chronic	0 (0.0%)	0 (0.0%)	
Steroids 2 weeks prior to RSV diagnosis, n (%)			.16
Yes	5 (50.0%)	45 (27.8%)	
No	5 (50.0%)	117 (72.2%)	
IVIG 4 weeks before RSV diagnosis, n (%)			> .99
Yes	3 (30.0%)	58 (35.8%)	
No	7 (70.0%)	104 (64.2%)	
Palivizumab 4 weeks before RSV diagnosis, n (%)			> .99
Yes	0 (0.0%)	8 (4.9%)	
No	10 (100.0%)	154 (95.1%)	
Co-pathogen in blood, n (%)			.60
Yes	0 (0.0%)	19 (11.7%)	
No	10 (100.0%)	143 (88.3%)	
Co-pathogen in respiratory tract, n (%)			.49
Yes	4 (40.0%)	47 (29.0%)	
No	6 (60.0%)	115 (71.0%)	
Lowest ALC <300 μL prior to RSV diagnosis, n (%)	1 (10.0%)	38 (24.2%)	.46
Closest ALC <300 μL prior to RSV diagnosis, n (%)	2 (20.0%)	28 (17.6%)	.69
Lowest ANC <500 mm3 prior to RSV diagnosis, n (%)	1 (10.0%)	23 (14.5%)	> .99
Closest ANC <500 mm3 prior to RSV diagnosis, n (%)	1 (10.0%)	15 (9.4%)	> .99
Lowest monocyte <100 μL prior to RSV diagnosis, n (%)	1 (10.0%)	29 (18.5%)	.69
Closest monocyte <100 < μL prior to RSV diagnosis, n (%)	3 (30.0%)	19 (11.9%)	.13
Age (y) at RSV diagnosis, Median [Min, Max]	7.0 [2.0, 18.0]	6.0 [0.0, 19.0]	.80
Race, n (%)			.74
White	7 (70.0%)	95 (58.6%)	
Other or unknown	3 (30.0%)	67 (41.4%)	
Underlying diagnosis, n (%)			.044
Hematologic malignancy only	1 (10.0%)	74 (45.7%)	
Others^	9 (90.0%)	88 (54.3%)	
Organ failure, n (%)			> .99
Yes	0 (0.0%)	8 (4.9%)	
No	10 (100.0%)	154 (95.1%)	
HCT type, n (%)			.70
Autologous	3 (30.0%)	36 (22.2%)	
Allogeneic	7 (70.0%)	126 (77.8%)	
Myeloablative conditioning, n (%)			.73
Yes	8 (80.0%)	115 (71.0%)	
No	2 (20.0%)	47 (29.0%)	
Acute GvHD Grade, n (%)			.20
1	0 (0.0%)	6 (18.2%)	
2	0 (0.0%)	16 (48.5%)	
3	1 (50.0%)	8 (24.2%)	
4	1 (50.0%)	3 (9.1%)	
GvHD/Rejection prophylaxis or treatment, n (%)			> .99
Yes	5 (50.0%)	87 (53.7%)	
No	5 (50.0%)	75 (46.3%)	
B cell depletion 6 months before RSV infection, n (%)			.60
Yes	0 (0.0%)	18 (11.1%)	
No	10 (100.0%)	144 (88.9%)	

^ Others include rare solid tumor (2), hematologic disorder (2), multiple conditions (2), brain tumor (1), inherited immunodeficiency (1), and other underlying diagnosis (1).

Abbreviations: ALC, absolute lymphocyte count; ANC, absolute neutrophil count; ATG, antithymocyte globulin; CMV, cytomegalovirus; GvHD, graft-versus-host disease; HCT, hematopoietic cell transplantation; IVIG, intravenous immunoglobulin; LRTI, lower respiratory tract infection; RSV, respiratory syncytial virus; URTI, upper respiratory tract infection.

## DISCUSSION

This multicenter cohort study provides a pediatric-specific assessment of RSV severity after HCT, identifying risk factors for clinically meaningful respiratory compromise, particularly new or increased supplemental oxygen requirement and LRTI. This oxygen outcome occurred in one-fifth of patients and served as a robust marker of disease severity. Invasive mechanical ventilation and RSV-attributable mortality were uncommon, limiting further risk factor analysis.

A key contribution of this study is the identification of risk factors for new or increased supplemental oxygen requirement.^33^^,^^34^ This outcome captured clinically significant lower respiratory involvement and aligns with broader pediatric RSV experience in which respiratory support serves as a stable measure of disease severity.^30^^,^^35^^,^^36^ Female sex, age < 5 years, chronic lung disease, and neutropenia were independently associated with this outcome, indicating that severe disease was more closely linked to host factors than transplant-specific characteristics.

Markers of impaired immune recovery were consistently associated with severe outcomes. Using both nadir and values closest to RSV diagnosis, lower ALC, ANC, and monocyte counts showed similar directional associations with RSV-related hospitalization, the oxygen outcome, and mortality. Neutropenia demonstrated the strongest and most reproducible association with severe RSV outcomes and mirrored findings from predominantly adult HCT cohorts.^8^^,^^9^ While neutropenia may reflect delayed immune recovery in many pediatric HCT recipients, it may also result from subsequent therapies or intercurrent illness. Therefore, neutropenia should be interpreted as a clinically relevant marker of host immune status at the time of RSV infection rather than a direct measure of immune recovery. Shorter time from transplant was associated with the oxygen outcome in univariate analysis but was not retained in multivariable models after inclusion of laboratory markers of immune recovery. This finding suggests that these laboratory measures may more directly reflect susceptibility to severe RSV disease than time from transplantation alone. Chronic lung disease has been associated with respiratory support and mortality in pediatric HCT recipients with viral respiratory infections, including RSV.^32^ Its role in adult HCT cohorts, which comprise most RSV data, remains less clearly defined and warrants further study.

Age was also an important determinant of severe RSV disease. Although younger children in the general population are at highest risk for severe RSV-related lower respiratory tract disease, it has been unclear whether this vulnerability persists among pediatric HCT recipients. In our cohort, age ≥ 5 years was independently associated with a lower risk of the oxygen outcome, whereas children <2 and 2–4 years had comparable risk. During the study period, palivizumab prophylaxis was recommended for children younger than two years,^21^^,^^22^^,^^37^^,^^38^ and a protective trend was observed in univariable analysis. However, the small number of recipients and the strong correlation between age < 2 years and palivizumab receipt precluded disentangling the independent effects of age and prophylaxis. As preventive strategies expand, careful evaluation of age-related risk and RSV monoclonal antibody prophylaxis remains essential, as these factors distinguish pediatric from adult HCT populations and carry important practical implications.^10^^,^^30^^,^^39^ These findings underscore the need for pediatric-specific risk assessment tools rather than reliance on adult-derived scoring systems.^21^^,^^22^^,^^37^^,^^38^

Female sex was associated with higher odds of the oxygen outcome and should be interpreted cautiously. Although sex-related differences in respiratory virus severity have been described in children,^40^ their role in RSV severity after pediatric HCT is unclear. This association may reflect unmeasured confounding rather than a biological effect and warrants evaluation in larger prospective cohorts.

Risk factors for RSV-related hospitalization differed from those for the oxygen outcome and included recent exposure to T cell–depleting agents (ATG or alemtuzumab) and a graft source other than blood stem cells. However, RSV-related hospitalization in this population is influenced by institutional practice patterns, complicating its interpretation as a marker of severity. Recent exposure to T-cell–depleting therapies was associated with the oxygen outcome in univariable analyses but did not retain statistical significance in multivariable models. Although biologically plausible, its impact on RSV-related outcomes has not been well characterized in prior pediatric and adult studies. In contrast, steroid exposure within the two weeks preceding RSV diagnosis was not associated with RSV-related outcomes, despite steroid use being an established risk factor for poor outcomes in predominantly adult HCT recipients with respiratory viral infections.^8^^,^^9^^,^^28^^,^^41^

We attempted to identify factors associated with LRTI, but none were significant in multivariable models. Similar challenges have been described in other pediatric immunocompromised cohorts.^39^^,^^42^^,^^43^ Variability in radiographic interpretation and documentation likely contributed to these findings. Progression from URTI to LRTI was uncommon, limiting assessment of risk factors for progression.^28^^,^^44^

This study’s strengths include its multicenter design, diverse population, and harmonized data abstraction. Prior work in immunocompromised children has included small numbers of transplant recipients^10^ or focused primarily on adults.^5^^,^^6^^,^^8^ However, despite the large number of cases, the sample size did not permit multivariable analyses for some outcomes, including invasive mechanical ventilation and mortality. Although treatment variables were collected descriptively, post-diagnosis therapies were not included in the primary multivariable models because of the potential for confounding by indication. Given its retrospective design, unrecognized or unmeasured confounders cannot be excluded. In addition, RSV-related hospitalization should be interpreted cautiously because the predefined study variable did not distinguish new RSV-attributable admissions from continued or prolonged hospitalization among children who were already hospitalized when RSV was diagnosed. Consequently, we could not determine the incremental impact of RSV on hospitalization in these patients. Prospective studies are needed to validate these findings and refine pediatric-specific prediction models.

In conclusion, this multicenter study identifies risk factors for severe RSV outcomes in pediatric HCT recipients, particularly new or increased supplemental oxygen requirement, and highlights the roles of markers of impaired immune recovery, younger age, and preexisting pulmonary disease. These findings support more targeted prevention strategies informed by age and immune recovery. Notably, children aged 2–4 years had risk profiles comparable to those younger than 2 years, suggesting prevention strategies may warrant consideration beyond the youngest age group.

## Supplementary Material

R1Hijano_PIDTRAN_Supplementary_Results_piag092

## Abbreviations

RSV: respiratory syncytial virus
HCT: hematopoietic cell transplant
URTI: upper respiratory tract infection
LRTI: lower respiratory tract infection
EMR: electronic medical record
ALL: acute lymphoblastic leukemia
LRA: logistic regression analysis
PO: per os (by mouth)
IRB: institutional review board
PIDTRAN: pediatric infectious diseases transplant network
ALC: absolute lymphocyte count
ANC: absolute neutrophil count
OR: odds ratio
CI: confidence interval
LASSO: least absolute shrinkage and selection operator
ATG: anti-thymocyte globulin
