# Supplementary material for: Burden and Risk Factors for Severe Respiratory Syncytial Virus Infection among Pediatric Hematopoietic Cell Transplant Recipients
Source: J Pediatric Infect Dis Soc. 2026 Sep 10;15(9):piag092. doi: 10.1093/jpids/piag092 (PMC13641079; doi:10.1093/jpids/piag092)
Supplement: Supplementary file 1 [file r1hijano_pidtran_supplementary_results_piag092.docx]

**Supplementary Results**

**Supplementary Table 1. Distribution of respiratory and blood co-pathogens identified within 48 hours of RSV diagnosis among pediatric HCT recipients**

| Respiratory co-pathogens (n = 63) | |
| --- | --- |
| Pathogen category | **No. of patients** |
| Adenovirus | 14 |
| Rhinovirus/Enterovirus | 24 |
| Coronavirus | 16 |
| Influenza A/B | 7 |
| Human metapneumovirus | 3 |
| Parainfluenza virus | 3 |
| Bacterial pathogens* | 5 |
| Fungal pathogens† | 3 |
| Blood co-pathogens (n = 25) | |
| Pathogen category | **No. of patients** |
| CMV | 8 |
| EBV | 8 |
| Adenovirus | 3 |
| BK virus | 2 |
| Bacterial bloodstream pathogens‡ | 11 |
| Fungal pathogens† | 2 |

Abbreviations: CMV, cytomegalovirus; EBV, Epstein-Barr virus; HCT, hematopoietic cell transplantation; RSV, respiratory syncytial virus. *Bacterial respiratory pathogens included organisms such as *Haemophilus parainfluenzae*, vancomycin-resistant enterococci, and other bacteria identified from respiratory specimens. †Fungal pathogens included *Candida*, *Fusarium*, and other fungal organisms documented in respiratory or blood specimens. ‡Bacterial bloodstream pathogens included *Enterobacter cloacae*, *Enterococcus* species, *Staphylococcus aureus*, viridans group streptococci, and other documented bloodstream bacterial infections. **Patients could have more than one co-pathogen; therefore, category totals exceed the number of patients with respiratory (n = 63) or blood (n = 25) co-pathogens.**

**Supplementary Table 2. Maximum level of respiratory support among pediatric HCT recipients requiring supplemental oxygen following RSV infection (N = 43)**

| Maximum level of respiratory support | N (%) |
| --- | --- |
| Low-flow oxygen (nasal cannula, mask, blow-by, face tent, trach collar, venturi mask) | 26 (60.5%) |
| High-flow nasal cannula | 6 (14.0%) |
| CPAP/BiPAP | 1 (2.3%) |
| Invasive mechanical ventilation | 8 (18.6%) |
| Unknown | 2 (4.7%) |
| Total | **43 (100%)** |

**Abbreviations:** BiPAP, bilevel positive airway pressure; CPAP, continuous positive airway pressure; HCT, hematopoietic cell transplantation; RSV, respiratory syncytial virus.

**Supplementary Table 3. Invasive mechanical ventilation among pediatric HCT recipients**

| Characteristic | Yes (N = 8) | No (N = 193) | P-value |
| --- | --- | --- | --- |
| Age group, n (%) |  |  | 0.009 |
| <2y | 4 (50.0%) | 18 (9.3%) |  |
| 2-4y | 1 (12.5%) | 51 (26.4%) |  |
| ≥5 y | 3 (37.5%) | 124 (64.2%) |  |
| Sex, n (%) |  |  | 0.15 |
| Male | 2 (25.0%) | 107 (55.4%) |  |
| Female | 6 (75.0%) | 86 (44.6%) |  |
| Number of transplantations, n (%) |  |  | >0.99 |
| 1 | 7 (87.5%) | 163 (84.5%) |  |
| 2 or 3 | 1 (12.5%) | 30 (15.5%) |  |
| Allo-HCT type, n (%) |  |  | 0.77 |
| Matched sibling donor | 1 (16.7%) | 38 (25.5%) |  |
| Matched unrelated donor | 4 (66.7%) | 50 (33.6%) |  |
| Mismatched unrelated donor | 0 (0.0%) | 9 (6.0%) |  |
| Haploidentical | 0 (0.0%) | 21 (14.1%) |  |
| Cord/related or unrelated | 1 (16.7%) | 29 (19.5%) |  |
| Other | 0 (0.0%) | 2 (1.3%) |  |
| Source of transplant, n (%) |  |  | 0.074 |
| Cord blood | 3 (37.5%) | 35 (18.1%) |  |
| Bone marrow | 5 (62.5%) | 95 (49.2%) |  |
| Peripheral blood | 0 (0.0%) | 63 (32.6%) |  |
| Induction medications for transplantation, n (%) |  |  | 0.047 |
| ATG | 4 (50.0%) | 36 (18.7%) |  |
| Alemtuzumab | 1 (12.5%) | 14 (7.3%) |  |
| No/Other | 3 (37.5%) | 143 (74.1%) |  |
| CMV serostatus, n (%) |  |  | 0.4 |
| D+/R+ | 1 (20.0%) | 52 (32.7%) |  |
| D+/R- | 0 (0.0%) | 14 (8.8%) |  |
| D-/R+ | 0 (0.0%) | 31 (19.5%) |  |
| D-/R- | 4 (80.0%) | 62 (39.0%) |  |
| Co-morbidities, n (%) |  |  | 0.44 |
| Yes | 4 (50.0%) | 62 (32.1%) |  |
| No | 4 (50.0%) | 131 (67.9%) |  |
| Chronic lung disease, n (%) |  |  | 0.52 |
| Yes | 1 (12.5%) | 16 (8.5%) |  |
| No | 7 (87.5%) | 173 (91.5%) |  |
| GvHD diagnosis, n (%) |  |  | 0.68 |
| None | 6 (75.0%) | 136 (70.5%) |  |
| Acute | 1 (12.5%) | 43 (22.3%) |  |
| Chronic | 1 (12.5%) | 14 (7.3%) |  |
| Both-Acute and Chronic | 0 (0.0%) | 0 (0.0%) |  |
| Steroids 2 weeks prior to RSV diagnosis, n (%) |  |  | 0.25 |
| Yes | 4 (50.0%) | 57 (29.5%) |  |
| No | 4 (50.0%) | 136 (70.5%) |  |
| IVIG 4 weeks before RSV diagnosis, n (%) |  |  | 0.13 |
| Yes | 5 (62.5%) | 66 (34.2%) |  |
| No | 3 (37.5%) | 127 (65.8%) |  |
| Palivizumab 4 weeks before RSV diagnosis, n (%) |  |  | >0.99 |
| Yes | 0 (0.0%) | 8 (4.1%) |  |
| No | 8 (100.0%) | 185 (95.9%) |  |
| Co-pathogen in blood, n (%) |  |  | 0.23 |
| Yes | 2 (25.0%) | 21 (10.9%) |  |
| No | 6 (75.0%) | 172 (89.1%) |  |
| Co-pathogen in respiratory tract, n (%) |  |  | 0.11 |
| Yes | 5 (62.5%) | 57 (29.5%) |  |
| No | 3 (37.5%) | 136 (70.5%) |  |
| Lowest ALC <300 µL prior to RSV diagnosis, n (%) | 4 (50.0%) | 46 (24.3%) | 0.11 |
| Closest ALC <300 µL prior to RSV diagnosis, n (%) | 3 (37.5%) | 36 (18.8%) | 0.19 |
| Lowest ANC <500 mm3 prior to RSV diagnosis, n (%) | 4 (50.0%) | 29 (15.2%) | 0.027 |
| Closest ANC <500 mm3 prior to RSV diagnosis, n (%) | 3 (37.5%) | 21 (10.9%) | 0.057 |
| Lowest monocyte <100 µL prior to RSV diagnosis, n (%) | 2 (25.0%) | 35 (18.5%) | 0.65 |
| Closest monocyte <100 µL prior to RSV diagnosis, n (%) | 2 (25.0%) | 27 (14.1%) | 0.33 |
| Age (y) at RSV diagnosis, Median [Min, Max] | 2.0 [0.0, 18.0] | 6.0 [0.0, 19.0] | 0.14 |
| Race, n (%) |  |  | >0.99 |
| White | 5 (62.5%) | 113 (58.5%) |  |
| Other or unknown | 3 (37.5%) | 80 (41.5%) |  |
| Underlying diagnosis, n (%) |  |  | 0.14 |
| Hematologic malignancy only | 1 (12.5%) | 84 (43.5%) |  |
| Others | 7 (87.5%) | 109 (56.5%) |  |
| Organ failure, n (%) |  |  | 0.34 |
| Yes | 1 (12.5%) | 9 (4.7%) |  |
| No | 7 (87.5%) | 184 (95.3%) |  |
| HCT type, n (%) |  |  | >0.99 |
| Autologous | 2 (25.0%) | 44 (22.8%) |  |
| Allogeneic | 6 (75.0%) | 149 (77.2%) |  |
| Myeloablative conditioning, n (%) |  |  | >0.99 |
| Yes | 6 (75.0%) | 141 (73.1%) |  |
| No | 2 (25.0%) | 52 (26.9%) |  |
| Acute GvHD Grade, n (%) |  |  | 0.091 |
| 1 | 0 (0.0%) | 6 (14.0%) |  |
| 2 | 0 (0.0%) | 22 (51.2%) |  |
| 3 | 0 (0.0%) | 12 (27.9%) |  |
| 4 | 1 (100.0%) | 3 (7.0%) |  |
| GvHD/Rejection prophylaxis or treatment, n (%) |  |  | 0.47 |
| Yes | 6 (75.0%) | 107 (55.4%) |  |
| No | 2 (25.0%) | 86 (44.6%) |  |
| B cell depletion 6 months before RSV infection, n (%) |  |  | >0.99 |
| Yes | 0 (0.0%) | 18 (9.4%) |  |
| No | 8 (100.0%) | 174 (90.6%) |  |

^ Others include brain tumor, solid tumor, hematologic disorder, inherited immunodeficiency, other underlying diagnoses, and multiple conditions.

**Abbreviations:** ALC, absolute lymphocyte count; ANC, absolute neutrophil count; ATG, antithymocyte globulin; CMV, cytomegalovirus; GvHD, graft-versus-host disease; HCT, hematopoietic cell transplantation; IVIG, intravenous immunoglobulin; RSV, respiratory syncytial virus.

**Supplementary Table 4. Death within 90 days of RSV diagnosis among pediatric HCT recipients**

| Characteristic | Yes (N = 11) | No (N = 193) | P-value |
| --- | --- | --- | --- |
| Age group, n (%) |  |  | 0.009 |
| <2y | 4 (36.4%) | 18 (9.3%) |  |
| 2-4y | 4 (36.4%) | 49 (25.4%) |  |
| ≥5y | 3 (27.3%) | 126 (65.3%) |  |
| Sex, n (%) |  |  | 0.12 |
| Male | 3 (27.3%) | 108 (56.0%) |  |
| Female | 8 (72.7%) | 85 (44.0%) |  |
| Number of transplantations, n (%) |  |  | 0.38 |
| 1 | 8 (72.7%) | 165 (85.5%) |  |
| 2 or 3 | 3 (27.3%) | 28 (14.5%) |  |
| Allo-HCT type, n (%) |  |  | 0.31 |
| Matched sibling donor | 0 (0.0%) | 39 (26.4%) |  |
| Matched unrelated donor | 4 (44.4%) | 50 (33.8%) |  |
| Mismatched unrelated donor | 1 (11.1%) | 8 (5.4%) |  |
| Haploidentical | 1 (11.1%) | 21 (14.2%) |  |
| Cord/related or unrelated | 3 (33.3%) | 28 (18.9%) |  |
| Other | 0 (0.0%) | 2 (1.4%) |  |
| Source of transplant, n (%) |  |  | 0.003 |
| Cord blood | 6 (54.5%) | 33 (17.1%) |  |
| Bone marrow | 1 (9.1%) | 100 (51.8%) |  |
| Peripheral blood | 4 (36.4%) | 60 (31.1%) |  |
| Induction medications for transplantation, n (%) |  |  | 0.24 |
| ATG | 4 (36.4%) | 36 (18.7%) |  |
| Alemtuzumab | 1 (9.1%) | 14 (7.3%) |  |
| No/Other | 6 (54.5%) | 143 (74.1%) |  |
| CMV serostatus, n (%) |  |  | 0.68 |
| D+/R+ | 2 (22.2%) | 52 (33.1%) |  |
| D+/R- | 1 (11.1%) | 13 (8.3%) |  |
| D-/R+ | 1 (11.1%) | 30 (19.1%) |  |
| D-/R- | 5 (55.6%) | 62 (39.5%) |  |
| Co-morbidities, n (%) |  |  | 0.001 |
| Yes | 9 (81.8%) | 59 (30.6%) |  |
| No | 2 (18.2%) | 134 (69.4%) |  |
| Chronic lung disease, n (%) |  |  | 0.60 |
| Yes | 0 (0.0%) | 17 (9.0%) |  |
| No | 11 (100.0%) | 171 (91.0%) |  |
| GvHD diagnosis, n (%) |  |  | 0.17 |
| None | 6 (54.5%) | 138 (71.5%) |  |
| Acute | 5 (45.5%) | 40 (20.7%) |  |
| Chronic | 0 (0.0%) | 15 (7.8%) |  |
| Both-Acute and Chronic | 0 (0.0%) | 0 (0.0%) |  |
| Steroids 2 weeks prior to RSV diagnosis, n (%) |  |  | 0.093 |
| Yes | 6 (54.5%) | 56 (29.0%) |  |
| No | 5 (45.5%) | 137 (71.0%) |  |
| IVIG 4 weeks before RSV diagnosis, n (%) |  |  | 0.20 |
| Yes | 6 (54.5%) | 66 (34.2%) |  |
| No | 5 (45.5%) | 127 (65.8%) |  |
| Palivizumab 4 weeks before RSV diagnosis, n (%) |  |  | >0.99 |
| Yes | 0 (0.0%) | 8 (4.1%) |  |
| No | 11 (100.0%) | 185 (95.9%) |  |
| Co-pathogen in blood, n (%) |  |  | 0.005 |
| Yes | 5 (45.5%) | 20 (10.4%) |  |
| No | 6 (54.5%) | 173 (89.6%) |  |
| Co-pathogen in respiratory tract, n (%) |  |  | 0.32 |
| Yes | 5 (45.5%) | 58 (30.1%) |  |
| No | 6 (54.5%) | 135 (69.9%) |  |
| Lowest ALC <300 µL prior to RSV diagnosis vs. ≥300 µL , n (%) | 8 (72.7%) | 43 (22.9%) | 0.001 |
| Closest ALC <300 µL prior to RSV diagnosis vs. ≥300 µL , n (%) | 6 (54.5%) | 34 (17.9%) | 0.009 |
| Lowest ANC <500 mm3 prior to RSV diagnosis, n (%) | 6 (54.5%) | 28 (14.7%) | 0.004 |
| Closest ANC <500 mm3 prior to RSV diagnosis, n (%) | 3 (27.3%) | 21 (11.0%) | 0.13 |
| Lowest monocyte <100 µL prior to RSV diagnosis, n (%) | 7 (63.6%) | 31 (16.5%) | 0.001 |
| Closest monocyte <100 µL prior to RSV diagnosis, n (%) | 3 (27.3%) | 27 (14.2%) | 0.21 |
| Age (y) at RSV diagnosis, Median [Min, Max] | 2.0 [0.0, 18.0] | 6.0 [0.0, 19.0] | 0.020 |
| Race, n (%) |  |  | 0.53 |
| White | 5 (45.5%) | 114 (59.1%) |  |
| Other or unknown | 6 (54.5%) | 79 (40.9%) |  |
| Underlying diagnosis, n (%) |  |  | 0.36 |
| Hematologic malignancy only | 3 (27.3%) | 83 (43.0%) |  |
| Others | 8 (72.7%) | 110 (57.0%) |  |
| Organ failure, n (%) |  |  | 0.001 |
| Yes | 4 (36.4%) | 7 (3.6%) |  |
| No | 7 (63.6%) | 186 (96.4%) |  |
| HCT type, n (%) |  |  | >0.99 |
| Autologous | 2 (18.2%) | 45 (23.3%) |  |
| Allogeneic | 9 (81.8%) | 148 (76.7%) |  |
| Myeloablative conditioning, n (%) |  |  | 0.73 |
| Yes | 9 (81.8%) | 140 (72.5%) |  |
| No | 2 (18.2%) | 53 (27.5%) |  |
| Acute GvHD Grade, n (%) |  |  | 0.013 |
| 1 | 0 (0.0%) | 6 (15.0%) |  |
| 2 | 2 (40.0%) | 20 (50.0%) |  |
| 3 | 0 (0.0%) | 12 (30.0%) |  |
| 4 | 3 (60.0%) | 2 (5.0%) |  |
| GvHD/Rejection prophylaxis or treatment, n (%) |  |  | 0.35 |
| Yes | 8 (72.7%) | 106 (54.9%) |  |
| No | 3 (27.3%) | 87 (45.1%) |  |
| B cell depletion 6 months before RSV infection, n (%) |  |  | 0.6 |
| Yes | 0 (0.0%) | 18 (9.4%) |  |
| No | 11 (100.0%) | 174 (90.6%) |  |

**Abbreviations:** ALC, absolute lymphocyte count; ANC, absolute neutrophil count; ATG, antithymocyte globulin; CMV, cytomegalovirus; GvHD, graft-versus-host disease; HCT, hematopoietic cell transplantation; IVIG, intravenous immunoglobulin; RSV, respiratory syncytial virus.

**Supplementary Table 5. Risk factors for oxygen requirement among pediatric HCT recipients using dichotomized age categories (<5 vs ≥5 years).**

| Variable | Univariate | | Multivariable 1 | | Multivariable 2 | |
| --- | --- | --- | --- | --- | --- | --- |
|  | **Raw OR (95% CI)** | **P-value** | **Adjusted OR (95% CI)** | **P-value** | **Adjusted OR (95% CI)** | **P-value** |
| Age ≥5y vs. <5y | 0.41 (0.20, 0.81) | 0.011 | 0.37 (0.16, 0.83) | 0.016 | 0.36 (0.15, 0.82) | 0.016 |
| Female vs. Male | 1.88 (0.95, 3.73) | 0.071 | 3.80 (1.39, 10.40) | 0.009 | 3.65 (1.29, 10.32) | 0.014 |
| Allogeneic (matched) vs. Autologous | 0.51 (0.22, 1.17) | 0.112 |  |  |  |  |
| Allogeneic (other) vs. Autologous | 0.53 (0.21, 1.30) | 0.163 |  |  |  |  |
| Peripheral Blood vs. Others | 0.95 (0.46, 2.00) | 0.899 |  |  |  |  |
| ATG/Alemtuzumab vs. No/Other | 2.10 (1.02, 4.31) | 0.044 |  |  |  |  |
| CMV status: R+ vs R- | 0.80 (0.39, 1.63) | 0.536 |  |  |  |  |
| Number of transplantations (2 or 3 vs. 1) | 2.01 (0.83, 4.84) | 0.121 |  |  |  |  |
| Co-morbidities (Yes vs. No) | 2.03 (1.01, 4.06) | 0.045 | 1.93 (0.79, 4.70) | 0.147 | 2.20 (0.87, 5.56) | 0.095 |
| Chronic lung disease (Yes vs. No) | 2.92 (0.92, 9.23) | 0.068 | 4.17 (1.14, 15.28) | 0.031 | 4.31 (1.24, 15.00) | 0.022 |
| GvHD (Yes vs. No) | 0.90 (0.42, 1.92) | 0.792 |  |  |  |  |
| IVIG 4 weeks before RSV (Yes vs. No) | 1.81 (0.91, 3.60) | 0.093 |  |  |  |  |
| Palivizumab 4 weeks before RSV (Yes vs. No) | 0.52 (0.03, 8.21) | 0.643 |  |  |  |  |
| Steroids 2 weeks prior to RSV (Yes vs. No) | 1.13 (0.54, 2.34) | 0.749 |  |  |  |  |
| Co-pathogen in blood (Yes vs. No) | 1.20 (0.42, 3.47) | 0.732 |  |  |  |  |
| Co-pathogen in respiratory tract (Yes vs. No) | 1.84 (0.91, 3.72) | 0.089 | 2.39 (1.03, 5.55) | 0.043 |  |  |
| Days from transplant to RSV diagnosis | 0.996 (0.993, 0.999) | 0.018 |  |  |  |  |
| Lowest ALC prior to RSV diagnosis (<300 µL vs. ≥300 µL) | 2.44 (1.17, 5.08) | 0.017 |  |  |  |  |
| Lowest ANC prior to RSV diagnosis (<500 mm3 vs. ≥500 mm3) | 4.06 (1.79, 9.22) | <0.001 | 8.05 (2.63, 24.66) | <0.001 |  |  |
| Lowest monocyte prior to RSV diagnosis (<100 µL vs. ≥100 µL) | 2.00 (0.89, 4.51) | 0.094 |  |  |  |  |
| Closest ALC prior to RSV diagnosis (<300 µL vs. ≥300 µL) | 3.99 (1.84, 8.67) | <0.001 |  |  | 2.62 (0.83, 8.32) | 0.102 |
| Closest ANC prior to RSV diagnosis (<500 mm3 vs. ≥500 mm3) | 6.03 (2.31, 15.77) | <0.001 |  |  | 7.27 (1.39, 38.12) | 0.019 |
| Closest monocyte prior to RSV diagnosis (<100 µL vs. ≥100 µL) | 3.72 (1.57, 8.81) | 0.003 |  |  |  |  |

**Abbreviations:** ALC, absolute lymphocyte count; ANC, absolute neutrophil count; ATG, antithymocyte globulin; CI, confidence interval; CMV, cytomegalovirus; GvHD, graft-versus-host disease; IVIG, intravenous immunoglobulin; LASSO, least absolute shrinkage and selection operator; OR, odds ratio; RSV, respiratory syncytial virus.

**MV1:** Multivariable model using the lowest immune cell count prior to RSV diagnosis. **MV2:** Multivariable model using the immune cell count closest to RSV diagnosis. These models represent two prespecified approaches to evaluating immune reconstitution.

Variables with a p-value <0.10 in univariate analyses were included in the candidate pool for multivariable modeling, and final variable selection was performed using LASSO. Robust standard errors were computed using the sandwich estimator. A two-sided p-value <0.05 was considered statistically significant.

**Supplementary Table 6. RSV-related hospitalization rates by participating center among pediatric HCT recipients**

| Site | Total, N | Hospitalized, n | Hospitalized n/N (%) |
| --- | --- | --- | --- |
| University of Florida | 1 | 1 | 100 |
| The University of Chicago | 1 | 1 | 100 |
| Cincinnati Children's Hospital Medical Center | 21 | 18 | 85.7 |
| University of Minnesota Medical School | 16 | 13 | 81.2 |
| Nationwide Children's Hospital | 5 | 3 | 60 |
| The University of Alabama at Birmingham | 6 | 3 | 50 |
| Seattle Children's Hospital | 44 | 20 | 45.5 |
| Children's Hospital Colorado | 14 | 5 | 35.7 |
| Duke University | 9 | 3 | 33.3 |
| Emory University/CHOA | 25 | 8 | 32 |
| Northwestern University/Lurie Children's Hospital, Children's Hospital of Chicago | 8 | 2 | 25 |
| St Jude Children’s Research Hospital | 38 | 7 | 18.4 |
| Children's National Medical Center | 8 | 1 | 12.5 |
| MD Anderson | 5 | 0 | 0 |
| Cook Children's Medical Center | 2 | 0 | 0 |
| Wayne State/Children's Hospital of Michigan (Detroit) | 1 | 0 | 0 |

**Abbreviations:** HCT, hematopoietic cell transplantation; RSV, respiratory syncytial virus.

**Supplementary Table 7. RSV-related hospitalization among pediatric HCT recipients**

| Characteristic | Yes (N = 85) | No (N = 119) | P-value |
| --- | --- | --- | --- |
| Age group, n (%) |  |  | 0.64 |
| <2y | 11 (12.9%) | 11 (9.2%) |  |
| 2-4y | 23 (27.1%) | 30 (25.2%) |  |
| ≥5 y | 51 (60.0%) | 78 (65.5%) |  |
| Sex, n (%) |  |  | 0.15 |
| Male | 41 (48.2%) | 70 (58.8%) |  |
| Female | 44 (51.8%) | 49 (41.2%) |  |
| Number of transplantations, n (%) |  |  | 0.84 |
| 1 | 73 (85.9%) | 100 (84.0%) |  |
| 2 or 3 | 12 (14.1%) | 19 (16.0%) |  |
| Allo-HCT type, n (%) |  |  | 0.01 |
| Matched sibling donor | 14 (20.0%) | 25 (28.7%) |  |
| Matched unrelated donor | 32 (45.7%) | 22 (25.3%) |  |
| Mismatched unrelated donor | 3 (4.3%) | 6 (6.9%) |  |
| Haploidentical | 4 (5.7%) | 18 (20.7%) |  |
| Cord/related or unrelated | 16 (22.9%) | 15 (17.2%) |  |
| Other | 1 (1.4%) | 1 (1.1%) |  |
| Source of transplant, n (%) |  |  | <0.001 |
| Cord blood | 18 (21.2%) | 21 (17.6%) |  |
| Bone marrow | 53 (62.4%) | 48 (40.3%) |  |
| Peripheral blood | 14 (16.5%) | 50 (42.0%) |  |
| Induction medications for transplantation, n (%) |  |  | <0.001 |
| ATG | 26 (30.6%) | 14 (11.8%) |  |
| Alemtuzumab | 9 (10.6%) | 6 (5.0%) |  |
| No/Other | 50 (58.8%) | 99 (83.2%) |  |
| CMV serostatus, n (%) |  |  | 0.39 |
| D+/R+ | 19 (31.1%) | 35 (33.3%) |  |
| D+/R- | 5 (8.2%) | 9 (8.6%) |  |
| D-/R+ | 8 (13.1%) | 23 (21.9%) |  |
| D-/R- | 29 (47.5%) | 38 (36.2%) |  |
| Co-morbidities, n (%) |  |  | 0.051 |
| Yes | 35 (41.2%) | 33 (27.7%) |  |
| No | 50 (58.8%) | 86 (72.3%) |  |
| Chronic lung disease, n (%) |  |  | 0.018 |
| Yes | 12 (14.8%) | 5 (4.2%) |  |
| No | 69 (85.2%) | 113 (95.8%) |  |
| GvHD diagnosis, n (%) |  |  | 0.072 |
| None | 54 (63.5%) | 90 (75.6%) |  |
| Acute | 21 (24.7%) | 24 (20.2%) |  |
| Chronic | 10 (11.8%) | 5 (4.2%) |  |
| Both-Acute and Chronic | 0 (0.0%) | 0 (0.0%) |  |
| Steroids 2 weeks prior to RSV diagnosis, n (%) |  |  | 0.22 |
| Yes | 30 (35.3%) | 32 (26.9%) |  |
| No | 55 (64.7%) | 87 (73.1%) |  |
| IVIG 4 weeks before RSV diagnosis, n (%) |  |  | 0.011 |
| Yes | 39 (45.9%) | 33 (27.7%) |  |
| No | 46 (54.1%) | 86 (72.3%) |  |
| Palivizumab 4 weeks before RSV diagnosis, n (%) |  |  | >0.99 |
| Yes | 3 (3.5%) | 5 (4.2%) |  |
| No | 82 (96.5%) | 114 (95.8%) |  |
| Co-pathogen in blood, n (%) |  |  | 0.13 |
| Yes | 14 (16.5%) | 11 (9.2%) |  |
| No | 71 (83.5%) | 108 (90.8%) |  |
| Co-pathogen in respiratory tract, n (%) |  |  | >0.99 |
| Yes | 26 (30.6%) | 37 (31.1%) |  |
| No | 59 (69.4%) | 82 (68.9%) |  |
| Lowest ALC <300 µL prior to RSV diagnosis, n (%) | 24 (28.6%) | 27 (23.5%) | 0.42 |
| Closest ALC <300 µL prior to RSV diagnosis, n (%) | 19 (22.6%) | 21 (17.9%) | 0.47 |
| Lowest ANC <500 mm3 prior to RSV diagnosis, n (%) | 12 (14.3%) | 22 (18.8%) | 0.45 |
| Closest ANC <500 mm3 prior to RSV diagnosis, n (%) | 10 (11.9%) | 14 (11.9%) | >0.99 |
| Lowest monocyte <100 µL prior to RSV diagnosis, n (%) | 17 (20.2%) | 21 (18.3%) | 0.72 |
| Closest monocyte <100 µL prior to RSV diagnosis, n (%) | 15 (17.9%) | 15 (12.8%) | 0.33 |
| Age (y) at RSV diagnosis, Median [Min, Max] | 5.5 [0.0, 18.0] | 7.0 [0.0, 19.0] | 0.054 |
| Race, n (%) |  |  | 0.15 |
| White | 55 (64.7%) | 64 (53.8%) |  |
| Other or unknown | 30 (35.3%) | 55 (46.2%) |  |
| Underlying diagnosis, n (%) |  |  | 0.014 |
| Hematologic malignancy only | 27 (31.8%) | 59 (49.6%) |  |
| Others | 58 (68.2%) | 60 (50.4%) |  |
| Organ failure, n (%) |  |  | 0.37 |
| Yes | 3 (3.5%) | 8 (6.7%) |  |
| No | 82 (96.5%) | 111 (93.3%) |  |
| HCT type, n (%) |  |  | 0.13 |
| Autologous | 15 (17.6%) | 32 (26.9%) |  |
| Allogeneic | 70 (82.4%) | 87 (73.1%) |  |
| Myeloablative conditioning, n (%) |  |  | 0.078 |
| Yes | 68 (80.0%) | 81 (68.1%) |  |
| No | 17 (20.0%) | 38 (31.9%) |  |
| Acute GvHD Grade, n (%) |  |  | 0.85 |
| 1 | 2 (9.5%) | 4 (16.7%) |  |
| 2 | 11 (52.4%) | 11 (45.8%) |  |
| 3 | 5 (23.8%) | 7 (29.2%) |  |
| 4 | 3 (14.3%) | 2 (8.3%) |  |
| GvHD/Rejection prophylaxis or treatment, n (%) |  |  | 0.77 |
| Yes | 49 (57.6%) | 65 (54.6%) |  |
| No | 36 (42.4%) | 54 (45.4%) |  |
| B cell depletion 6 months before RSV infection, n (%) |  |  | 0.22 |
| Yes | 10 (11.9%) | 8 (6.7%) |  |
| No | 74 (88.1%) | 111 (93.3%) |  |

**Abbreviations:** ALC, absolute lymphocyte count; ANC, absolute neutrophil count; ATG, antithymocyte globulin; CMV, cytomegalovirus; GvHD, graft-versus-host disease; HCT, hematopoietic cell transplantation; IVIG, intravenous immunoglobulin; RSV, respiratory syncytial virus.

**Supplementary Table 8. Logistic regression analysis of risk factors associated with RSV-related hospitalization among pediatric HCT recipients using lowest and closest immune cell counts prior to RSV diagnosis.**

|  | Univariate | | Multivariable 1 | | Multivariable 2 | |
| --- | --- | --- | --- | --- | --- | --- |
| Variable | Raw OR (95% CI) | P-value | Adjusted OR (95% CI) | P-value | Adjusted OR (95% CI) | P-value |
| Age 2-4y vs. <2y | 0.77 (0.27, 2.20) | 0.621 |  |  |  |  |
| Age ≥5y vs. <2y | 0.65 (0.25, 1.72) | 0.388 |  |  |  |  |
| Female vs. Male | 1.53 (0.87, 2.69) | 0.137 |  |  |  |  |
| Allogeneic (matched) vs. Autologous | 2.09 (0.99, 4.40) | 0.053 |  |  |  |  |
| Allogeneic (other) vs. Autologous | 1.28 (0.57, 2.86) | 0.548 |  |  |  |  |
| Peripheral Blood vs. Others | 0.27 (0.14, 0.54) | <0.001 | 0.36 (0.18, 0.75) | 0.006 | 0.36 (0.18, 0.75) | 0.006 |
| ATG/Alemtuzumab vs. No/Other | 3.46 (1.80, 6.67) | <0.001 | 2.53 (1.27, 5.04) | 0.008 | 2.53 (1.27, 5.04) | 0.008 |
| CMV status: R+ vs R- | 0.75 (0.42, 1.36) | 0.348 |  |  |  |  |
| Number of transplantations (2 or 3 vs. 1) | 0.87 (0.38, 1.97) | 0.729 |  |  |  |  |
| Co-morbidities (Yes vs. No) | 1.82 (1.01, 3.31) | 0.047 |  |  |  |  |
| Chronic lung disease (Yes vs. No) | 3.93 (1.17, 13.25) | 0.027 |  |  |  |  |
| GvHD (Yes vs. No) | 1.78 (0.96, 3.30) | 0.066 |  |  |  |  |
| IVIG 4 weeks before RSV (Yes vs. No) | 2.21 (1.22, 3.99) | 0.008 |  |  |  |  |
| Palivizumab 4 weeks before RSV (Yes vs. No) | 0.83 (0.13, 5.53) | 0.851 |  |  |  |  |
| Steroids 2 weeks prior to RSV (Yes vs. No) | 1.48 (0.81, 2.72) | 0.204 |  |  |  |  |
| Co-pathogen in blood (Yes vs. No) | 1.94 (0.78, 4.80) | 0.154 |  |  |  |  |
| Co-pathogen in respiratory tract (Yes vs. No) | 0.98 (0.53, 1.80) | 0.939 |  |  |  |  |
| Lowest ALC prior to RSV diagnosis (<300 µL vs. ≥300 µL) | 1.30 (0.68, 2.50) | 0.424 |  |  |  |  |
| Lowest ANC prior to RSV diagnosis (<500 mm3 vs. ≥500 mm3) | 0.72 (0.32, 1.60) | 0.418 |  |  |  |  |
| Lowest monocyte prior to RSV diagnosis (<100 µL vs. ≥100 µL) | 1.14 (0.55, 2.36) | 0.734 |  |  |  |  |
| Closest ALC prior to RSV diagnosis (<300 µL vs. ≥300 µL) | 1.34 (0.65, 2.73) | 0.426 |  |  |  |  |
| Closest ANC prior to RSV diagnosis (<500 mm3 vs. ≥500 mm3) | 1.00 (0.40, 2.55) | 0.994 |  |  |  |  |
| Closest monocyte prior to RSV diagnosis (<100 µL vs. ≥100 µL) | 1.48 (0.65, 3.35) | 0.348 |  |  |  |  |

**Abbreviations:** ALC, absolute lymphocyte count; ANC, absolute neutrophil count; ATG, antithymocyte globulin; CI, confidence interval; CMV, cytomegalovirus; GvHD, graft-versus-host disease; IVIG, intravenous immunoglobulin; LASSO, least absolute shrinkage and selection operator; OR, odds ratio; RSV, respiratory syncytial virus.

**MV1:** Multivariable model using the lowest immune cell count prior to RSV diagnosis. **MV2:** Multivariable model using the immune cell count closest to RSV diagnosis. These models represent two prespecified approaches to evaluating immune reconstitution.

Variables with a p-value <0.10 in univariate analyses were included in the candidate pool for multivariable modeling, and final variable selection was performed using LASSO. Robust standard errors were computed using the sandwich estimator. A two-sided p-value <0.05 was considered statistically significant.

**Supplementary Table 9. Sensitivity analysis of risk factors for lower respiratory tract infection (LRTI), defined as LRTI at diagnosis or progression from URTI to LRTI, among pediatric HCT recipients.**

| Characteristic | URTI (N = 162) | LRTI (N = 38) | P-value |
| --- | --- | --- | --- |
| Age group, n (%) |  |  | 0.38 |
| <2y | 19 (11.7%) | 3 (7.9%) |  |
| 2-4y | 38 (23.5%) | 13 (34.2%) |  |
| ≥5y | 105 (64.8%) | 22 (57.9%) |  |
| Sex, n (%) |  |  | 0.15 |
| Male | 84 (51.9%) | 25 (65.8%) |  |
| Female | 78 (48.1%) | 13 (34.2%) |  |
| Number of transplantations, n (%) |  |  | >0.99 |
| 1 | 137 (84.6%) | 33 (86.8%) |  |
| 2 or 3 | 25 (15.4%) | 5 (13.2%) |  |
| Allo-HCT type, n (%) |  |  | 0.95 |
| Matched sibling donor | 32 (25.4%) | 6 (21.4%) |  |
| Matched unrelated donor | 41 (32.5%) | 11 (39.3%) |  |
| Mismatched unrelated donor | 7 (5.6%) | 2 (7.1%) |  |
| Haploidentical | 19 (15.1%) | 3 (10.7%) |  |
| Cord/related or unrelated | 25 (19.8%) | 6 (21.4%) |  |
| Other | 2 (1.6%) | 0 (0.0%) |  |
| Source of transplant, n (%) |  |  | 0.92 |
| Cord blood | 31 (19.1%) | 8 (21.1%) |  |
| Bone marrow | 79 (48.8%) | 19 (50.0%) |  |
| Peripheral blood | 52 (32.1%) | 11 (28.9%) |  |
| Induction medications for transplantation, n (%) |  |  | 0.019 |
| ATG | 26 (16.0%) | 14 (36.8%) |  |
| Alemtuzumab | 13 (8.0%) | 2 (5.3%) |  |
| No/Other | 123 (75.9%) | 22 (57.9%) |  |
| CMV serostatus, n (%) |  |  | 0.85 |
| D+/R+ | 41 (31.5%) | 13 (39.4%) |  |
| D+/R- | 12 (9.2%) | 2 (6.1%) |  |
| D-/R+ | 25 (19.2%) | 5 (15.2%) |  |
| D-/R- | 52 (40.0%) | 13 (39.4%) |  |
| Co-morbidities, n (%) |  |  | 0.12 |
| Yes | 49 (30.2%) | 17 (44.7%) |  |
| No | 113 (69.8%) | 21 (55.3%) |  |
| Chronic lung disease, n (%) |  |  | 0.75 |
| Yes | 15 (9.5%) | 2 (5.4%) |  |
| No | 143 (90.5%) | 35 (94.6%) |  |
| GvHD diagnosis, n (%) |  |  | 0.75 |
| None | 116 (71.6%) | 26 (68.4%) |  |
| Acute | 33 (20.4%) | 10 (26.3%) |  |
| Chronic | 13 (8.0%) | 2 (5.3%) |  |
| Both-Acute and Chronic | 0 (0.0%) | 0 (0.0%) |  |
| Steroids 2 weeks prior to RSV diagnosis, n (%) |  |  | 0.17 |
| Yes | 45 (27.8%) | 15 (39.5%) |  |
| No | 117 (72.2%) | 23 (60.5%) |  |
| IVIG 4 weeks before RSV diagnosis, n (%) |  |  | 0.71 |
| Yes | 58 (35.8%) | 12 (31.6%) |  |
| No | 104 (64.2%) | 26 (68.4%) |  |
| Palivizumab 4 weeks before RSV diagnosis, n (%) |  |  | 0.36 |
| Yes | 8 (4.9%) | 0 (0.0%) |  |
| No | 154 (95.1%) | 38 (100.0%) |  |
| Co-pathogen in blood, n (%) |  |  | >0.99 |
| Yes | 19 (11.7%) | 4 (10.5%) |  |
| No | 143 (88.3%) | 34 (89.5%) |  |
| Co-pathogen in respiratory tract, n (%) |  |  | 0.24 |
| Yes | 47 (29.0%) | 15 (39.5%) |  |
| No | 115 (71.0%) | 23 (60.5%) |  |
| Lowest ALC <300 µL prior to RSV diagnosis, n (%) | 38 (24.2%) | 12 (31.6%) | 0.41 |
| Closest ALC <300 µL prior to RSV diagnosis, n (%) | 28 (17.6%) | 11 (28.9%) | 0.12 |
| Lowest ANC <500 mm3 prior to RSV diagnosis, n (%) | 23 (14.5%) | 10 (26.3%) | 0.092 |
| Closest ANC <500 mm3 prior to RSV diagnosis, n (%) | 15 (9.4%) | 8 (21.1%) | 0.052 |
| Lowest monocyte <100 µL prior to RSV diagnosis, n (%) | 29 (18.5%) | 8 (21.1%) | 0.82 |
| Closest monocyte <100 µL prior to RSV diagnosis, n (%) | 19 (11.9%) | 10 (26.3%) | 0.039 |
| Age (y) at RSV diagnosis, Median [Min, Max] | 6.0 [0.0, 19.0] | 7.0 [1.0, 18.0] | 0.97 |
| Race, n (%) |  |  | >0.99 |
| White | 95 (58.6%) | 22 (57.9%) |  |
| Other or unknown | 67 (41.4%) | 16 (42.1%) |  |
| Underlying diagnosis, n (%) |  |  | 0.044 |
| Hematologic malignancy only | 74 (45.7%) | 10 (26.3%) |  |
| Others | 88 (54.3%) | 28 (73.7%) |  |
| Organ failure, n (%) |  |  | 0.44 |
| Yes | 8 (4.9%) | 3 (7.9%) |  |
| No | 154 (95.1%) | 35 (92.1%) |  |
| HCT type, n (%) |  |  | 0.67 |
| Autologous | 36 (22.2%) | 10 (26.3%) |  |
| Allogeneic | 126 (77.8%) | 28 (73.7%) |  |
| Myeloablative conditioning, n (%) |  |  | 0.42 |
| Yes | 115 (71.0%) | 30 (78.9%) |  |
| No | 47 (29.0%) | 8 (21.1%) |  |
| Acute GvHD Grade, n (%) |  |  | 0.49 |
| 1 | 6 (18.2%) | 0 (0.0%) |  |
| 2 | 16 (48.5%) | 5 (50.0%) |  |
| 3 | 8 (24.2%) | 3 (30.0%) |  |
| 4 | 3 (9.1%) | 2 (20.0%) |  |
| GvHD/Rejection prophylaxis or treatment, n (%) |  |  | 0.37 |
| Yes | 87 (53.7%) | 24 (63.2%) |  |
| No | 75 (46.3%) | 14 (36.8%) |  |
| B cell depletion 6 months before RSV infection, n (%) |  |  | 0.028 |
| Yes | 18 (11.1%) | 0 (0.0%) |  |
| No | 144 (88.9%) | 37 (100.0%) |  |

**Abbreviations:** ALC, absolute lymphocyte count; ANC, absolute neutrophil count; ATG, antithymocyte globulin; CMV, cytomegalovirus; GvHD, graft-versus-host disease; HCT, hematopoietic cell transplantation; IVIG, intravenous immunoglobulin; LRTI, lower respiratory tract infection; RSV, respiratory syncytial virus; URTI, upper respiratory tract infection.

**Supplementary Table 10. Logistic regression analysis of risk factors associated with LRTI (at diagnosis or progression from URTI) among pediatric HCT recipients.**

|  | Univariate | | Multivariable 1 | | Multivariable 2 | |
| --- | --- | --- | --- | --- | --- | --- |
| Variable | Raw OR (95% CI) | P-value | Adjusted OR (95% CI) | P-value | Adjusted OR (95% CI) | P-value |
| Age 2-4y vs. <2y | 2.17 (0.51, 9.27) | 0.297 |  |  |  |  |
| Age ≥5y vs. <2y | 1.33 (0.33, 5.30) | 0.689 |  |  |  |  |
| Female vs. Male | 0.56 (0.27, 1.18) | 0.125 |  |  |  |  |
| Allogeneic (matched) vs. Autologous | 0.84 (0.35, 2.04) | 0.697 |  |  |  |  |
| Allogeneic (other) vs. Autologous | 0.75 (0.28, 1.97) | 0.555 |  |  |  |  |
| Peripheral Blood vs. Others | 0.86 (0.39, 1.88) | 0.710 |  |  |  |  |
| ATG/Alemtuzumab vs. No/Other | 2.29 (1.09, 4.84) | 0.029 | 2.01 (0.92, 4.39) | 0.081 | 1.94 (0.87, 4.30) | 0.104 |
| CMV status: R+ vs R- | 1.29 (0.62, 2.69) | 0.488 |  |  |  |  |
| Number of transplantations (2 or 3 vs. 1) | 0.83 (0.28, 2.46) | 0.737 |  |  |  |  |
| Co-morbidities (Yes vs. No) | 1.87 (0.90, 3.87) | 0.093 | 1.72 (0.81, 3.65) | 0.161 | 1.86 (0.84, 4.13) | 0.126 |
| Chronic lung disease (Yes vs. No) | 0.54 (0.10, 3.00) | 0.485 |  |  |  |  |
| GvHD (Yes vs. No) | 1.16 (0.54, 2.52) | 0.700 |  |  |  |  |
| IVIG 4 weeks before RSV (Yes vs. No) | 0.83 (0.39, 1.77) | 0.626 |  |  |  |  |
| Steroids 2 weeks prior to RSV (Yes vs. No) | 1.70 (0.81, 3.56) | 0.164 |  |  |  |  |
| Co-pathogen in blood (Yes vs. No) | 0.89 (0.26, 3.05) | 0.847 |  |  |  |  |
| Co-pathogen in respiratory tract (Yes vs. No) | 1.60 (0.76, 3.35) | 0.216 |  |  |  |  |
| Lowest ALC prior to RSV diagnosis (<300 µL vs. ≥300 µL) | 1.45 (0.66, 3.17) | 0.359 |  |  |  |  |
| Lowest ANC prior to RSV diagnosis (<500 mm3 vs. ≥500 mm3) | 2.11 (0.88, 5.08) | 0.095 | 1.80 (0.73, 4.45) | 0.200 |  |  |
| Lowest monocyte prior to RSV diagnosis (<100 µL vs. ≥100 µL) | 1.18 (0.48, 2.91) | 0.724 |  |  |  |  |
| Closest ALC prior to RSV diagnosis (<300 µL vs. ≥300 µL) | 1.91 (0.83, 4.38) | 0.128 |  |  |  |  |
| Closest ANC prior to RSV diagnosis (<500 mm3 vs. ≥500 mm3) | 2.58 (0.93, 7.13) | 0.068 |  |  | 1.75 (0.34, 9.12) | 0.505 |
| Closest monocyte prior to RSV diagnosis (<100 µL vs. ≥100 µL) | 2.63 (1.06, 6.53) | 0.037 |  |  | 1.65 (0.36, 7.48) | 0.518 |

**Abbreviations:** ALC, absolute lymphocyte count; ANC, absolute neutrophil count; ATG, antithymocyte globulin; CI, confidence interval; CMV, cytomegalovirus; GvHD, graft-versus-host disease; IVIG, intravenous immunoglobulin; LASSO, least absolute shrinkage and selection operator; LRTI, lower respiratory tract infection; OR, odds ratio; RSV, respiratory syncytial virus; URTI, upper respiratory tract infection.

**MV1:** Multivariable model using the lowest immune cell count prior to RSV diagnosis. **MV2:** Multivariable model using the immune cell count closest to RSV diagnosis. These models represent two prespecified approaches to evaluating immune reconstitution.

Variables with a p-value <0.10 in univariate analyses were included in the candidate pool for multivariable modeling, and final variable selection was performed using LASSO. Robust standard errors were computed using the sandwich estimator. A two-sided p-value <0.05 was considered statistically significant.
